# Impact of COVID-19 on radiology education in Europe: a survey by the ESR Radiology Trainees Forum (RTF)

**DOI:** 10.1186/s13244-021-01113-3

**Published:** 2021-11-09

**Authors:** European Society of Radiology (ESR), Michail E. Klontzas, Eoin O’Malley, Saif Afat, Viktoria Pozdniakova, Martina Pecoraro, Carlo Catalano, Minerva Becker, Martin Reim

**Affiliations:** European Society of Radiology (ESR), Am Gestade 1, 1010 Vienna, Austria

**Keywords:** Education, Radiology, COVID-19, SARS-CoV-2, Survey

## Abstract

**Background:**

The ongoing COVID-19 pandemic has significantly affected radiology services around the globe. The impact of the crisis on radiology education in Europe has yet to be determined, in order to identify measures to achieve optimal training of radiologists during pandemics. The aim of this survey was to evaluate the impact of the pandemic on young radiologist members of the European Society of Radiology (ESR).

**Methods:**

A survey consisting of 28 questions was developed and distributed using *SurveyMonkey* to all ESR European radiologist members in training. The survey sought to collect information on three main themes, ‘demographics’, ‘training level’ and ‘effects of COVID-19’. The responses were statistically analysed with the use of R programming using descriptive statistics.

**Results:**

A total of 249 responses from 34 countries were collected. Specific training on COVID-19 was not offered to 52.2% (130) of the participants. A total of 196 participants were not redeployed to other specialities but only 46.2% of institutions allowed residents to work from home. E-learning was offered at 43% of the departments and most participants (86.2%) were not allowed to switch from clinical work to research. A minority (n = 13) were suspended with (30.8%) or without salary (38.5%) or were forced to take vacation/yearly holiday leave (7.7%) or sick leave (23%). Almost half of the participants did not have access to personal protective equipment and a minority of them had their financial status affected.

**Conclusions:**

The ongoing SARS-CoV-2 outbreak has significantly affected all aspects of postgraduate radiology training across the ESR member countries.

**Supplementary Information:**

The online version contains supplementary material available at 10.1186/s13244-021-01113-3.

## Key points

- A survey evaluated the impact of COVID-19 on radiology education.
- The pandemic had an impact on all aspects of radiology training in Europe.
- Trainee health, financial status and work routine were affected by the crisis.

## Introduction

The global crisis caused by the ongoing COVID-19 pandemic has significantly affected health services around the globe. According to data released by the World Health Organisation in April 2021, the pandemic continues to significantly disrupt health services in the majority of countries, resulting in staff redeployment, facility closures, patient hesitance to seek medical help and postponement of elective surgery [1]. These disruptions have also affected radiology services, such as the need for additional post-examination equipment sterilization and the risk imposed by close contact of radiologists with a potentially contagious patient, especially during ultrasound and interventional radiology procedures [2].

Health service disruption has also affected medical education at undergraduate and postgraduate levels [3–7]. Direct patient contact during the time of training is critical for the successful education of competent medical practitioners. Radiology education at the resident level has been significantly affected by changes in health service administration during the pandemic. Reports from the United States of America highlight difficulties in resident recruitment [8], interaction with patients and a multitude of training aspects, including reduced supervision, limited research possibilities and almost eliminated didactic learning [4].

Radiology training in Europe differs compared to the rest of the world with regards to the training curriculum, the administration of postgraduate medical training and accreditation requirements. In addition, variability in COVID-19 burden across European countries has led to a great variety of measures for the containment of the pandemic, which have affected radiology services and radiology resident education to a different extent among the European Society of Radiology (ESR) member countries. Therefore, the Radiology Trainees Forum (RTF) of the ESR created a survey to collect information on the impact of the pandemic on the education of radiology residents. This manuscript presents the results of the survey which reveal the impact the pandemic has had on young radiologists who are members of the ESR. The importance of the results presented herein lies in the evaluation of the extent that COVID-19 has affected various aspects of radiology training. This will allow the identification of suitable measures that can be applied to ensure seamless resident training during this and future pandemics.

## Materials and methods

### Study design—participants

**Fig. 1:**
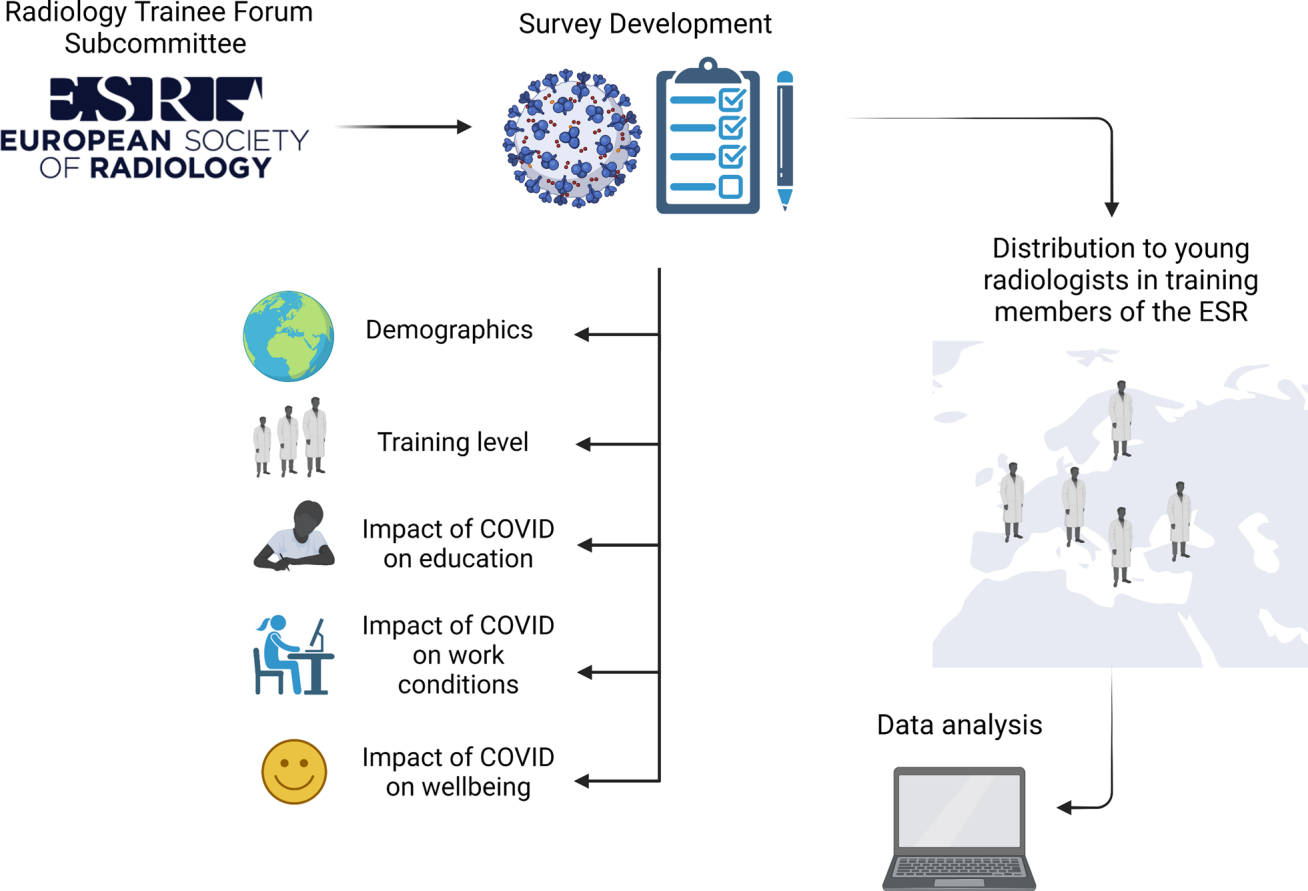
Flowchart describing the process of questionnaire development and data collection on the impact of COVID-19 on radiology training of young trainee members of the European Society of Radiology (ESR) (created with BioRender.com)

A survey consisting of 28 questions was developed by the Radiology Trainees Forum (RTF) to perform a comprehensive assessment of the effects of COVID-19 on radiology training in European Countries. ESR and RTF national delegates distributed the survey using *SurveyMonkey* to all ESR European radiologist members in training. The survey was distributed to radiology residents at all levels of training throughout the participating countries. Subspecialty fellows were also encouraged to participate, indicating their field of subspecialisation. Responses were automatically recorded. No institutional review board approval was required for this study (Fig. 1).

### Questionnaire structure

Questions covered three basic themes: ‘demographics’, ‘training level’ and ‘effects of COVID-19’. The ‘Demographics’ category included three questions on the country of residence, the age and sex of participants, whereas the ‘training level’ category included four questions on the year of training, the total duration of radiology training in the respective country, whether they are in a general radiology or subspecialty program and the type of training institution (university, regional, central or rural hospital). The remaining questions (21/28) were used to assess the impact of COVID-19 on various aspects of training, the modes of training delivery, the use of e-learning material, the type of work they conducted during the pandemic, the preparation of their institution to face a pandemic, the impact on their financial and work status and the supervision process. The detailed survey is provided as Additional file 1.

### Statistical analysis

Categorical responses to questionnaire questions were presented as frequencies and percentages and continuous variables were presented as mean ± standard deviations. Participant responses were mapped on the world map with the use of R programming (v 4.03, www.R-project.org).

## Results

**Fig. 2:**
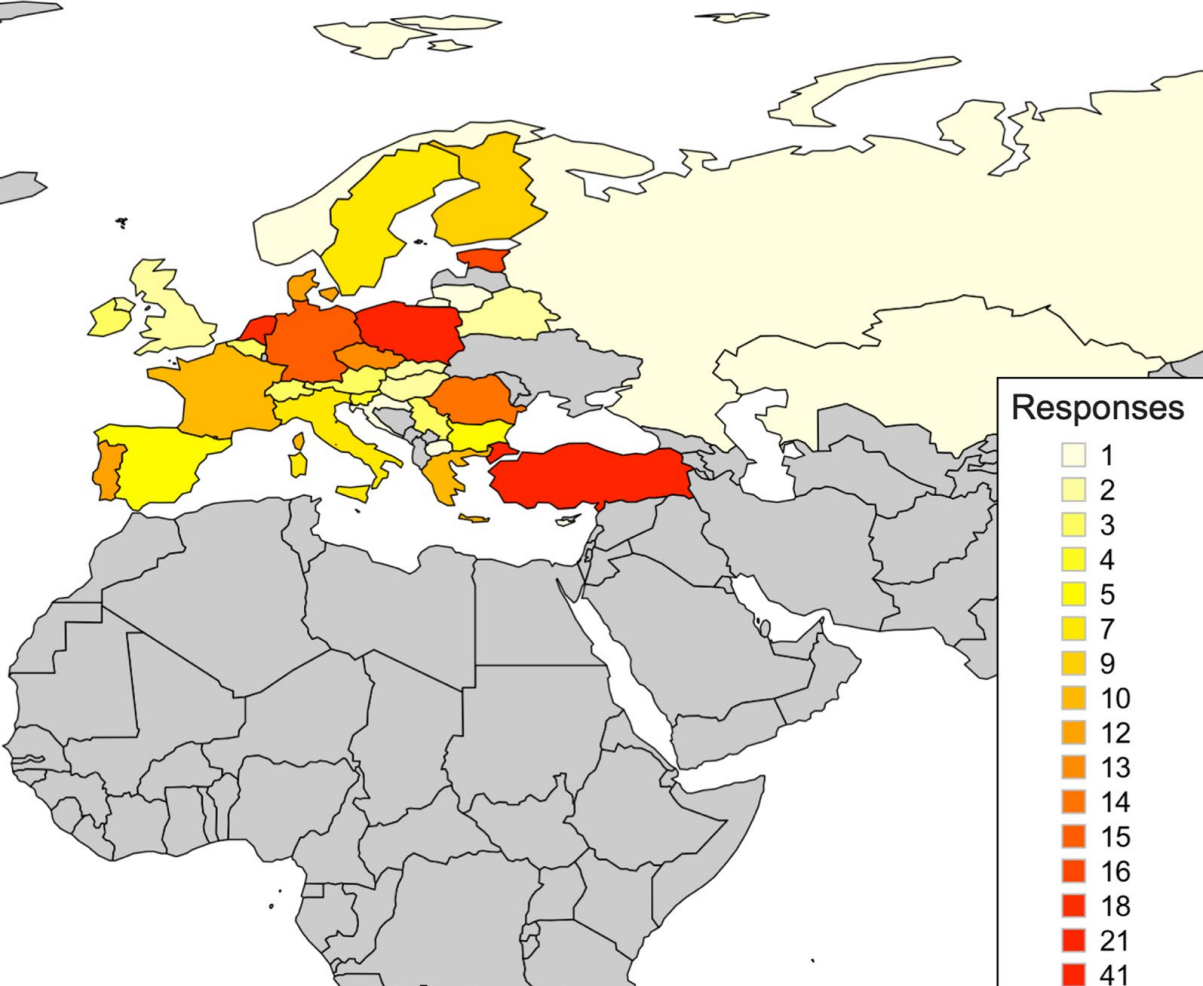
Map depicting the number of responses collected from each country member of the European Society of Radiology (ESR)

**Fig. 3:**
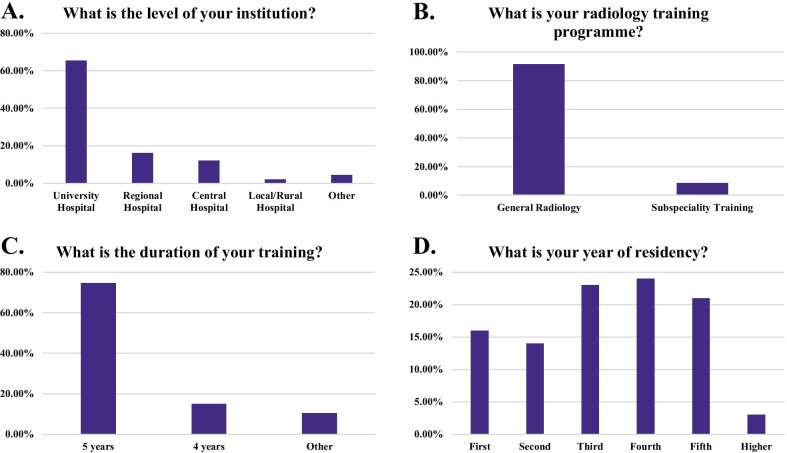
Responses to questions aiming to collect information about the type of training of young radiologists. The questions assessed the level of institution where the participants worked (**A**), the type of radiology training program (**B**), the duration of their training (**C**) and their year of residency (**D**)

A total of 249 resident responses were collected from 34 countries, with responses per country ranging between 1 and 41 (Fig. 2). Turkey and Poland were the countries that provided the most responses to the survey (41 and 21 responses respectively). Between 10 and 20 responses were received from the Netherlands, Estonia, Germany, Romania, Czech Republic, Denmark, Portugal, France and Greece. The mean ± SD age of participants was 31.3 ± 5 years (range 23–61) of whom 121 (48.6%) were male and 128 (51.4%) were female. Participants were uniformly spread along training years with 65.5% of them (163 residents) working at university hospitals in 5-year radiology programmes and were being trained on general radiology (91.6%—228 residents) (Fig. 3). The rest of the participants worked at regional hospitals (16.06%), central hospitals (12.05%), local/rural hospitals (2.01%) and other types of institutions (e.g., private institutions) (4.42%).

**Fig. 4:**
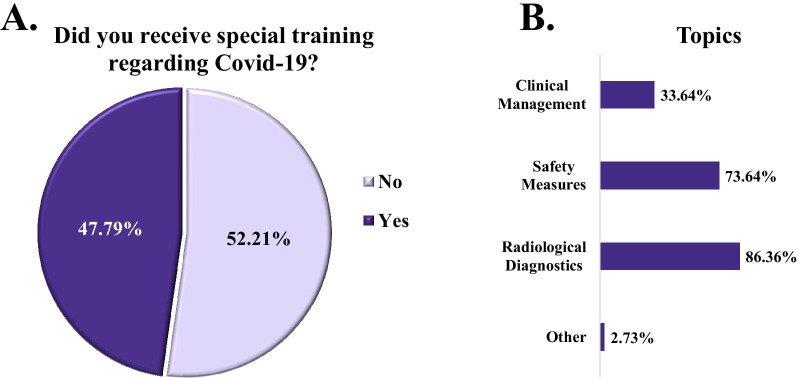
Responses to questions on the COVID-19 specific training offered to young radiologists. The questions assessed whether the trainees received special training (**A**) and what type of training it was (**B**)

Training on COVID-19 was not offered to 52.2% (130) of the participants. For those who received COVID-specific training, this was mainly focused (in 86.4% of cases) on the radiological diagnosis of the disease and the required safety measures (in 73.6% of cases) and less on the clinical management of the disease (in 33.6% of cases). A total of 172 (74.8%) participants received information on the clinical and radiological features of Sars-CoV-2 infection through online webinars and 152 (66.1%) of them reported that they were educated by international publications available online. A smaller number of participants (106—46.1%) mentioned that they were educated by European online publications, whereas 90 of them also read local or national papers. Finally, national or hospital guidelines provided information about the disease to 138 (60%) of the participants (Fig. 4).

With regards to the services that the participants were asked to provide during the pandemic, 85.2% (196 participants) replied that they were not redeployed to other specialities. The limited number of radiology trainees that were redeployed were asked to fill places in COVID-19 clinics, infectious disease departments, intensive care units and emergency departments and one participant was asked to refrain from patient contact due to ongoing pregnancy. The pandemic changed trainee views on the role of radiologists in 9.1% of cases with the majority of them reporting that they recognised the vital role of radiologists in the pandemic, with a limited number of trainees (3 out of 211 who replied to question 14) mentioning that radiological services were misused during the pandemic and that they felt that they became “service providers” without retaining any significant influence on patient management.

Radiology practices changed in most institutions during the pandemic by allowing more home office hours, reducing interpersonal contacts, providing online multidisciplinary meetings and following hospital safety measures. Only 46.2% of institutions allowed residents to use their home office in providing radiology services, provided they had appropriate secure access to the private network of the hospital and sufficient hardware to meet diagnostic requirements.

**Fig. 5:**
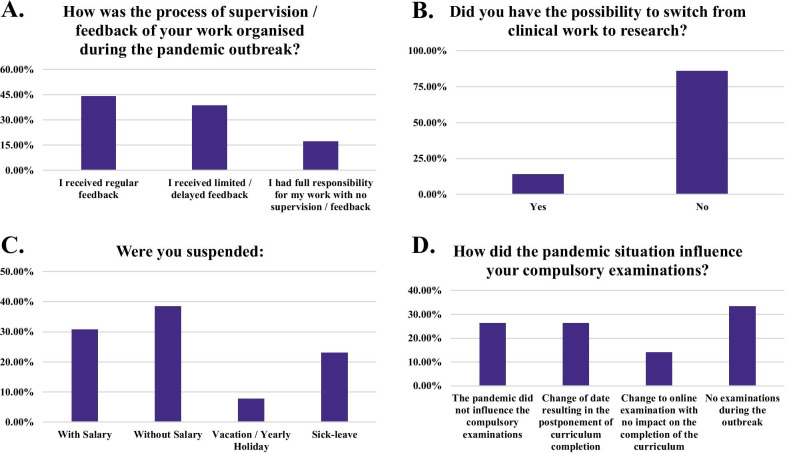
Responses to questions aiming to collect information about changes in radiological education during the pandemic. The questions assessed changes in supervision (**A**), the possibility to switch from clinical to research work (**B**), whether the trainees were suspended (**C**) and whether the outbreak affected compulsory examinations (**D**)

Training opportunities delivered in the form of e-learning were offered at 43% of the departments during the pandemic. 72.2% of the responding trainees were more or less satisfied with the usage of online learning possibilities. 48.3% of the participants rated the general usage of online education as a positive experience. Online training was considered an acceptable part of radiology training for 54.3% of participants, the majority of respondents specified that a certificate or Continuing Medical Education (CME) credits are needed. Importantly, supervision was greatly affected in participating countries because of COVID-19 with 38.7% of the participants reporting that they received limited or delayed feedback and 17.2% no feedback for their work. The vast majority of participants (86.2%) were not provided with the opportunity to switch from clinical work to research and 13 of them reported that they had been suspended because of COVID-19 either with (30.8%) or without salary (38.5%) or had been asked to take vacation/yearly holiday leave (7.7%) or sick leave (23%). In addition, the pandemic affected the compulsory residency examinations with 49 participants having the exam date postponed and subsequently the duration of their residency extended and 26 participants had the exams changed to an online format. 62 (33.3%) responders mentioned that no examinations were allowed during the outbreak (Fig. 5).

In terms of resident wellbeing during the outbreak, 51.6% of the participants have infected themselves or had colleagues infected by Sars-CoV-2. A small number of residents (14.1%) reported that they did not have access to the required safety equipment while dealing with COVID-19 patients and almost half of the participants (51.4%) did not have access to mental health resources during the pandemic. Finally, 7% of radiology trainees (n = 13) had their financial status affected by the pandemic because of their suspension from service.

## Discussion

This study aimed to evaluate the effect COVID-19 has had on radiology training throughout ESR member countries. The results of the survey demonstrated great variability within and between different countries and revealed that the pandemic significantly influenced supervision, teaching, examinations and the work environment of the participants. Importantly, the results showed that the outbreak has significantly impacted the quality of life of participants, affecting their health and their financial status.

Resident training was significantly affected over the course of the pandemic, with significant disruption to supervision. Almost half of our participants reported no access to online training and received limited or no feedback from their supervisors. Our results come to confirm the views of Alvin et al. [4] who foresaw that the current condition would be disadvantageous for supervision and proposed the application of remote meetings with supervisors to at least partly control the problem. A similar experience was reported from a Texas-based institution where interaction with supervising faculty has decreased either because of social distancing or because residents and fellows were deployed to other posts [3]. Our results show that the majority of trainees were not redeployed to other posts. Therefore, the effects on supervision could be mainly attributed to social distancing measures applied both for trainees and for their supervisors.

Theoretical radiology training during the outbreak has been primarily administered online. In some settings, this resulted in reduced resident learning time in contact with an experienced faculty member. A number of measures to electronically mitigate this problem have been proposed in literature such as adopting virtual readouts with the use of a camera so that senior radiologists can remotely supervise trainees [9]. Such proposals are hindered by the need to install costly hardware and PACS systems at the home office of young radiologists. However, the use of video call systems to host webinars and facilitate multidisciplinary team meetings has been proposed as a viable alternative [10]. At the same time, online learning enabled trainees to access the lectures held in other clinical institutions and created additional learning possibilities. Indeed, our participants reported a significant reliance on online education during the pandemic and the ESR and other radiology focused societies have enriched their webinar programs and modified the format of major conferences to facilitate the online dissemination of information while promoting social distancing. Such measures, however, cannot replace teaching for modalities that require patient contact (e.g. ultrasound and interventional procedures) [2] and cannot replace day-to-day supervision and case discussion with supervisors.

Exam delivery was disrupted to a variable extent across the countries of the participants, including where exams were cancelled for the whole duration of the outbreak. These changes have affected residents who were required to extend their training program and wait for qualifying exams. The core examination of the American Board of Radiology had been postponed for a significant time, delaying the process of graduation and posing hurdles for subsequent subspecialty training [4, 11]. Institutions like the EBR (European Board of Radiology) and the RCR (Royal College of Radiologists) have attempted to offer online proctored examinations as an alternative. The European Diploma in Radiology (EDiR) offered by the EBR stands as a viable alternative since it can be delivered by the local radiological societies and can also be delivered on-site for hospitals and institutions. In the past few months, several on-site EDiR e-examinations have been carried out, among others, in various cities in Croatia, France, Italy, Poland and countries outside Europe. Finally, the European Board of Radiology (EBR) has developed a software tool to conduct e-examinations in collaboration with the local national professional societies, scientific institutions and other speciality sections allowing them to organise their examinations locally for their residents. The first e-examinations were successfully held in September and October by the Consejo Mexicano de Radiología, allowing local residents to perform their board exams.

The well-being of young radiologists has been directly impacted by the SAR-CoV-2 outbreak. Approximately half of the participants reported that either themselves or their colleagues had been infected. In addition, some trainees were suspended, influencing their financial and psychological status and only half of them had access to mental health services. A recent study in the UK showed that 48% of radiology trainees wellbeing deteriorated during the pandemic [11] and trainees with ongoing student loans, health insurance and family-related financial obligations are expected to have their income reduced not only by the reduction in working hours [4] but also by the reduced reporting workload [4, 11] which could lead to delayed retirement and other financial hurdles. Potential solutions to this problem include offering paid leave to trainees who contract or become ill with COVID-19, to make sure that loan repayment can be extended and to make available mental health services for trainees with psychological struggles.

One limitation of our study is that the survey was not completed by residents in all ESR member countries. However, distribution of the survey was achieved through national radiological societies and the degree of participation was the maximum achievable given the ongoing pandemic situation. The size of the participant cohort was also affected by the voluntary nature of the survey. However, the validity of our results is indicated by abstract similarities with results presented from individual countries [11].

## Conclusion

The ongoing SARS-CoV-2 outbreak has significantly affected all aspects of postgraduate radiology training across the ESR member countries. Identification of these affected areas will assist in the development and implementation of mitigation measures and the preparation for potential future resurgences of SAR-CoV-2 or alternative similar situations.

## Supplementary Information

**Additional file 1.** Full survey.

## Data Availability

The full data is available upon reasonable request.

## Abbreviations

CME: Continuing Medical Education
COVID-19: Coronavirus Disease 2019
EBR: European Board of Radiology
EDiR: European Diploma in Radiology
ESR: European Society of Radiology
PACS: Picture Archiving and Communication System
RTF: Radiology Trainees Forum
Sars-CoV-2: Severe Acute Respiratory Syndrome Coronavirus 2
