# Supplementary material for: Impact of COVID-19 on radiology education in Europe: a survey by the ESR Radiology Trainees Forum (RTF)
Source: Insights Imaging. 2021 Nov 9;12:165. doi: 10.1186/s13244-021-01113-3 (PMC8576794; doi:10.1186/s13244-021-01113-3)
Supplement: Supplementary file 1 — Additional file 1. Full survey. [file 13244_2021_1113_MOESM1_ESM.pdf]

Questions:

1. Country of residency
    a. Free form answer
2. How old are you?
    a. Numerical answer
3. What is your sex?
    a. F/M
4. What is the level of your institution?
    a. University Hospital
    b. Regional Hospital
    c. Central Hospital
    d. Local/Rural Hospital/Other
5. What is your radiology training programme?
    a. General radiology
    b. Subspeciality training (please indicate the subspecialisation)
6. What is the duration of your training?
    a. 5 years
    b. 4 years
7. Please indicate your year of residency
    a. Numerical answer
8. Please specify how much did the Covid-19 influence the following aspects of your training:
    a. Supervision – scale 1 to 10
    b. Clinical session – scale 1 to 10
    c. Board meetings – scale 1 to 10
    d. Workload – scale 1 to 10
    e. Subspeciality training – scale 1 to 10
    f. Research – scale 1 to 10
9. Did you receive special training regarding the Covid infection?
    a. Yes/No
10. What were the fields covered in the Covid infection training?
    a. Clinical management
    b. Radiological diagnostics
    c. Safety measures
11. How did you learn about the Covid infection radiological and clinical features?
    a. Online webinars
    b. Hospital/national guidelines
    c. Online publications
        i. Local/National
        ii. European
        iii. International
12. How would you rate the preparations and reactions of your institution regarding the pandemic?
    a. Adaption of services – scale 1 to 10
    b. Resources (safety equipment, technical equipment) – scale 1 to 10
    c. Training of personnel – scale 1 to 10
13. Were you redeployed to other speciality services (clinical/non-clinical) during the pandemic?

   a. Yes/no
   b. If yes, please indicate the department/speciality
14. Did the Covid-19 pandemic changed your views about the role of radiologists?
    a. If yes, please specify
15. Are the e-learning modules available in your residency programme?
    a. If yes, please specify the online platform
16. How satisfied were you with the e-learning experience during the pandemic period?
    a. Scale 1 to 10 (1 indicating not satisfied at all and 10 indicating very satisfied, met my educational needs)
17. How would you rate the usage of online learning possibilities used in your institution?
    a. Scale 1-10
18. Does your institution accept online participation in conferences as part of your radiology training?
    a. Yes/no
    b. If yes, please indicate if any specific requirement has to be met (CME, certificate, etc.)
19. How your institution changed their practices in the light of possible second wave of Covid-19 infection?
    a. Free form question
20. Did you or your colleagues have possibility to use home office in providing radiology services?
    a. Yes/no
    b. If yes, please indicate if any specific requirement had to be met
21. How the process of supervision/feedback about your work was organised during the pandemic outbreak?
    a. I received regular feedback
    b. I received limited/delayed feedback
    c. I had full responsibility for my work with no supervision/feedback.
22. Did you have possibility to switch from clinical work to research?
    a. Yes/no
    b. If yes, was the time dedicated to research accepted as general monthly/weekly working hours?
23. How the pandemic situation influenced your compulsory examinations?
    a. The pandemic did not influence the compulsory examinations
    b. Change of date resulting postponement of completing the curriculum
    c. Change to online examination with no impact on the completion the curriculum
    d. No examinations during the outbreak
24. How the pandemic situation influenced your financial status?
    a. No influence
    b. I was suspended from the service

25. Were you suspended?

a) With salary?
b) Without salary?
c) Vacation/Yearly holiday
d) Sick-leave

26. Were you or your radiology colleagues infected at the workplace during the Covid-19 outbreak?

    c. Yes/no

27. Did you have access to required safety equipment while dealing with Covid + patients?
    a. Yes/no

28. Were hospital mental health resources available to the trainees during the pandemic?
    a. Yes/No
